# LncRNA HOTTIP as a diagnostic biomarker for acute respiratory distress syndrome in patients with sepsis and to predict the short-term clinical outcome: a case-control study

**DOI:** 10.1186/s12871-024-02405-z

**Published:** 2024-01-18

**Authors:** Weitao Shi, Wang Zhu, Jiani Yu, Yingjun Shi, Yuliang Zhao

**Affiliations:** 1 Department of Critical Care Medicine, The Affiliated Xuzhou Municipal Hospital of Xuzhou Medical University (The First People’s Hospital of Xuzhou), Xuzhou, Jiangsu Province 221000 China; 2 Department of Rheumatology and Immunology, The Affiliated Xuzhou Municipal Hospital of Xuzhou Medical University (The First People’s Hospital of Xuzhou), Xuzhou, Jiangsu Province 221000 China

**Keywords:** Sepsis, HOTTIP, ARDS, Diagnostic, Predicts

## Abstract

**Background:**

The present research aims to investigate the clinical diagnostic value of LncRNA HOXA distal transcript antisense RNA (HOTTIP) in acute respiratory distress syndrome (ARDS) of sepsis and its predictive significance for mortality.

**Methods:**

One hundred eighteenth patients with sepsis and 96 healthy individuals were enrolled. RT-qPCR to examine HOTTIP levels. The incidence of ARDS and death was recorded. The diagnostic significance of HOTTIP in sepsis ARDS was examined using ROC and logistic regression analysis. The correlation between HOTTIP and disease severity was evaluated using Pearson’s coefficients. Kaplan-Meier analysis and COX regression were employed to examine the predictive significance of mortality. Validation of HOTTIP target miRNA by dual-luciferase assay.

**Results:**

HOTTIP was persistently up-regulated in patients with ARDS sepsis than in patients without ARDS patients (*P* < 0.05). HOTTIP was a risk factor for the development of ARDS, which could be diagnosed in ARDS patients from non-ARDS patients (AUC = 0.847). Both the SOFA score (*r* = 0.6793) and the APACHE II score (*r* = 0.6384) were positively correlated with the HOTTIP levels. Furthermore, serum HOTTIP was an independent predictor of short-term mortality (HR = 4.813. 95%CI: 1.471–15.750, *P* = 0.009) and noticeably predicted the occurrence of short-term death (log rank = 0.020). miR-574-5p, a target miRNA for HOTTIP, was reduced in patients with sepsis ARDS and negatively correlated with HOTTIP.

**Conclusions:**

The presence of HOTTIP serves as a diagnostic biomarker for the occurrence of ARDS, exhibits correlation with disease severity, and provides predictive value of short-term mortality in sepsis patients. HOTTIP may be involved in ARDS progression by targeting miR-574-5p.

**Supplementary Information:**

The online version contains supplementary material available at 10.1186/s12871-024-02405-z.

## Background

The occurrence of sepsis, a dysfunctional host response triggered by infection, is prevalent in clinical emergencies and is often accompanied by multiorgan failure and immune dysfunction [1]. More than 18 million people suffer from sepsis and cause almost nearly 250,000 deaths each year [2]. Acute respiratory distress syndrome (ARDS) is one of the poorest and most serious complications of sepsis and is the result of systemic inflammation, uncontrolled cytokines, and other causes of pulmonary dysfunction [3]. The prevalence of ARDS in patients with sepsis reaches up to 25% [4]. Early identification and assessment of changes in patients with sepsis combined with ARDS have been shown by clinical studies to be crucial to improve their prognosis.

Dysregulation of long noncoding RNAs (LncRNAs) was identified as the culprit in the pathogenesis of ARDS in sepsis. For example, silencing of LncRNA-ASLC12002 eliminates the epithelial-mesenchymal transition in sepsis-induced ARDS [2]. SNHG16 in patients with ARDS was significantly lower compared to nonARDS patients, and elevated levels of SNHG16 were independently associated with a lower incidence of ARDS [5]. The increase in THRIL significantly differentiated ARDS from nonARDS in patients with sepsis and was positively correlated with severity in patients with sepsis [6]. Thus, LncRNAs are associated with ARDS in sepsis. LncRNA HOXA distal transcript antisense RNA (HOTTIP) on chromosome 7q15.2 was characterized as an inflammation-associated gene. HOTTIP overexpression serves as a diagnostic biomarker of acute gouty arthritis and is associated with excessive secretion of inflammatory factors [7]. Presistent down-regulation of HOTTIP reduces airway inflammation in asthma [8]. Persistent down-regulation of HOTTIP expression effectively attenuates inflammation induced by high glucose and up-regulation of fibrillar-associated proteins in diabetic nephropathy [9]. In particular, ARDS is characterized by a rapid fibroproliferative response in addition to early inflammation. Pulmonary fibrosis is believed to be the main cause of a poor prognosis in patients with ARDS due to sepsis [2], while Li et al. in 2021 found that silencing HOTTIP alleviated pulmonary fibrosis [10]. Furthermore, elevated HOTTP was also observed in patients with sepsis and was associated with their cardiac dysfunction. However, the potential function of HOTTIP in sepsis-induced ARDS remains unclear.

Based on the above information, we hypothesize that HOTTIP plays a crucial function in the ARDS of sepsis, and we focus on its diagnostic and prognostic significance for ARDS patients.

## Methods

### Study design

Hospital based unmatched case control study design was employed. And the trial was conducted in accordance with the STROBE Statement checklist of items that should be included in reports of case-control studies (Supplementary materials 1).

### Sample size

The prevalence of sepsis with ARDS is about 25% [2, 11], while the prevalence of ARDS alone is about 9.5% [12]. According to the sample size calculation formula of the case-control study with an unmatched design [13, 14]: $$n=2\times$$$$\frac{p \left(1-p\right){\left({U}_{\alpha }+{U}_{\beta }\right)}^{2}}{{\left({p}_{1}-{p}_{2}\right)}^{2}}$$, a two-sided test was used with values of α = 0.05 and β = 0.20, U_α_ = 1.96 (from Z table) at type I error of 5%, U_β_= 0.84 (from Z table) at 80% power, p_1_ = prevalence in case group (p_1_ = 25), p2 = prevalence in control group (p2 = 9.5), p = pooled prevalence = [p_1_+p_2_]/2. Therefore, a minimum sample size of 94 participants per group was required, and we included a strict minimum sample size in this unmatched design case-control study in the control group but included more than the minimum number of patients in the patient group to the best of our ability.

### Participants in this research

One hundred eighteenth patients with sepsis admitted to the First People’s Hospital of Xuzhou from January 2019 to June 2020 were included. Eligible patients were enrolled consecutively for this one and a half-year period. The following criteria were met for enrolment: a) diagnosis as sepsis according to the 3rd International consensus definitions of sepsis; b) Age ≥ 18 years; c) admission to the hospital in the last 24 h. Furthermore, the following patients were excluded: a) patients who had received immunosuppressive therapy in the last 6 months; b) chronic organ failure; c) complicated with severe cardiac, liver, and renal impairment; d) death within 24 h after admission; e) patients with incomplete or missing clinical data. In addition, a control group of 94 healthy volunteers from the physical examination center was enrolled. They were matched in age and gender to patients with sepsis, had no recent immunosuppressive medication, no history of sepsis, and all had normal physical examination parameters. A flow chart diagram of patient inclusion can be found in the supplementary figure (Supplementary material 2).

This study was carried out in accordance with the principles of the Declaration of Helsinki. Approval (No. xyyl [2019] 012) was granted by the Ethics Committee of The First People’s Hospital of Xuzhou. Written informed consent was obtained from all subjects.

### Specimen collection

Record demographic characteristics of subjects, including age, gender, and body mass index (BMI) after admission. Biological indicators such as scr, WBC, and CRP were also analyzed. The APACHE II score and the SOFA score, indicators of the severity of sepsis and the severity of organ failure impairment, were assessed within 24 h after admission. Blood was collected from subjects on the day of admission, while controls were collected at enrollment. Blood samples were centrifuged and the upper serum was stored at -80℃.

### Quantitative real-time polymerase chain reaction (RT-qPCR)

RT-qPCR was used to quantify HOTTIP expression levels in patients with sepsis (including Non-ARDS and ARDS) and controls. The Easypure miRNA kit was utilized to isolate total serum RNA and examine its quality on a NanoDrop 1000 spectrophotometer. 2 µg RNA was synthesized into cDNA using the TranscScript Two-Step RT-PCR superMix kit on a PCR amplifier. Then, the SYBR Green qPCR Master Mix Kit reagent, cDNA, primers, and RNase Free H_2_O were added to the EP tubes and mixed to perform RT-qPCR amplification reactions in the CFX96 real-time PCR system. GAPDH and U6 served as internal control for LncRNA and miRNA, quantified by the 2^−ΔΔCt^ method and calculated after three independent replicate experiments.

### ARDS assessment

ARDS onset was promptly monitored and assessed according to the Berlin definition [15], which included: a) acute onset with new or aggravated respiratory symptoms within 1 week; b) chest imaging shows diffuse infiltrative shadows in both lungs that cannot be fully interpreted as exudates, atelectasis, masses; c) The origin of edema and respiratory failure cannot be explained by heart failure or excessive fluid input.

### Treatment and follow-up program

After admission, the patients received standard treatment and were resuscitated. The experiment began with the patient’s diagnosis of sepsis and ended with short-term follow-up of the patient over 28 days, recording the number of days from admission to death or last visit. The cumulative survival probability was calculated and the predictive power of HOTTIP for clinical outcomes was examined.

### Target miRNA prediction and validation

The online databases LncBook and DIANA predicted the target miRNAs of HOTTIP analyzed the overlapping miRNAs using Venn diagrams and found that miR-574-5p. The wild-type (WT) and mutant (MUT) sequences of HOTTIP containing the miR-574-5p binding site were amplified and inserted into the luciferase vector pmirGLO to form the recombinant plasmids HOTTIP-WT and HOTTIP-MUT, respectively. 293T cells were inoculated in 48-well plates. The miR-NC and miR-574-5p mimic (RiboBio) were cotransfected with HOTTIP-WT and HOTTIP-MUT, respectively, and the cells were lysed after 48 h. Luciferase activity was detected via the dual luciferase reporter gene assay system (Promega).

### Gene Ontology (GO) and Kyoto Encyclopedia of genes and genomes (KEGG) pathway analyses

The TargetScan, miRWalk, EVmiRNA, CancerMIRNome, and ENCORI databases predicted target mRNA for has-miR-574-5p, respectively. Additionally, the CTD database (https://ctdbase.org/) analyzes sepsis as well as genes related to ARDS. Venn diagrams were performed to analyze overlapping genes. Subsequently, the overlapping genes were entered into the bioinformatics platform, and “H.sapiens” was selected for the annotation of the GO and the enrichment analysis of the KEGG pathway, which uses the KEGG mapper onlie service tool provided by Kanehisa Laboratories (citation guidelines: www.kegg.jp/kegg/kegg1.html) [16–18].

### Statistical analysis

Data were presented as mean ± SD after three independent experiments. Data analysis and chart visualization were performed on SPSS 23.0 or GraphPad Prism 9.0 software. Data were compared between two groups using Student’s T-test and differences between multiple groups were analyzed using one-way and two-way ANOVA followed by Tukey’s post-hot test. The receiver operating characteristic (ROC) was carried out to examine the diagnostic significance (sensitivity and specificity). Log-rank test of Kapan-Meier curves to explore the predictive significance on short-term survival outcomes. *P* < 0.05 is a statistically significant difference.

## Results

### Demographic and clinicopathological data of the patients with sepsis

A total of 118 patients with sepsis were enrolled; their mean age was 54.43 ± 10.07 years, of which 55.93% were male. Primary sites of infection included 41 (34.75%) abdominal infections, 27 (22.88%) respiratory infections, 23 (19.49%) skin and soft tissue infections, 12 (10.17%) bloodstream infections, 6 (5.08%) CNS infections, and 9 (7.63%) other infections. Additionally, the median values of the biochemical parameters Scr, Albumin, WBC, CRP and PCT were 1.9 (1.4, 2.7) mg/dL, 27.2 (23.8, 30.7) g/L, 17.5 (12.6, 27.3) ×10^9^/L, 91.96 (60.88, 143.81) mg/L, 15.4 (8.9, 21.8) ng/mL. The inflammatory factors IL-1β, IL-6, and TNF-α were 13.8 (8.8, 20.2) pg/mL, 70.9 (45.2, 111.9) pg/mL and 188.1 (146.6, 233.8) g/mL. Detailed information is recorded in Table 1.

**Table 1:** Comparison of the baseline data of patients with sepsis

Parameters	Total sepsis patients (*n* = 118)
**Demographics**	
Age (year), mean ± SD	54.43 ± 10.07
Gender, n (%)	
Female	52 (44.07)
Male	66 (55.93)
BMI (kg/m^2^), mean ± SD	25.36 ± 2.73
Smoke, n (%)	43 (36.44)
**Complications**	
COPD, n (%)	24 (20.34)
Cardiomyopathy, n (%)	57 (48.31)
Chronic kidney failure, n (%)	19 (16.10)
Cirrhosis, n (%)	27 (22.88)
**Primary infection site**	
Abdominal infection, n (%)	41 (34.75)
Respiratory infection, n (%)	27 (22.88)
Skin and soft tissue infection, n (%)	23 (19.49)
Bloodstream infection, n (%)	12 (10.17)
CNS infection, n (%)	6 (5.08)
Other infection, n (%)	9 (7.63)
**Primary organism**	
G^−^, n (%)	65 (55.08)
G^+^, n (%)	26 (22.03)
Anaerobes, n (%)	13 (11.02)
Fungus, n (%)	7 (5.93)
Mycoplasmas, n (%)	5 (4.24)
Total culture negative, n (%)	23 (19.49)
**Biochemical indexes**	
Scr (mg/dL), median (IQR)	1.9 (1.4, 2.7)
Albumin, (g/L), median (IQR)	27.2 (23.8, 30.7)
WBC (×10^9^/L), median (IQR)	17.5 (12.6, 27.3)
CRP (mg/L), median (IQR)	91.93 (60.88, 143.81)
PCT (ng/mL), median (IQR)	15.4 (8.9, 21.8)
Inflammatory cytokines	
IL-1β (pg/mL)	13.8 (8.8, 20.2)
IL-6 (pg/mL)	70.9 (45.2, 111.9)
TNF-α (pg/mL)	188.1 (146.6, 233.8)
**Disease severity**	
APACHE II score, median (IQR)	13.0 (9.0, 18.0)
SOFA score, median (IQR)	6.0 (5.0, 9.0)

*BMI* Body mass index, *COPD* Chronic obstructive pulmonary disease, *CNS* Central nervous system, *APACHE* Acute physiology and chronic health evaluation, *SOFA* Sequential organ failure assessment, *Scr* Serum creatinine, *WBC* White blood cells, *CRP* C-reactive protein, *PCT* Procalcitonin, *TNF-α* Tumor necrosis factor-α, *IL* Interleukin

### Elevated serum HOTTIP is a diagnostic biomarker for sepsis

**Fig. 1:**
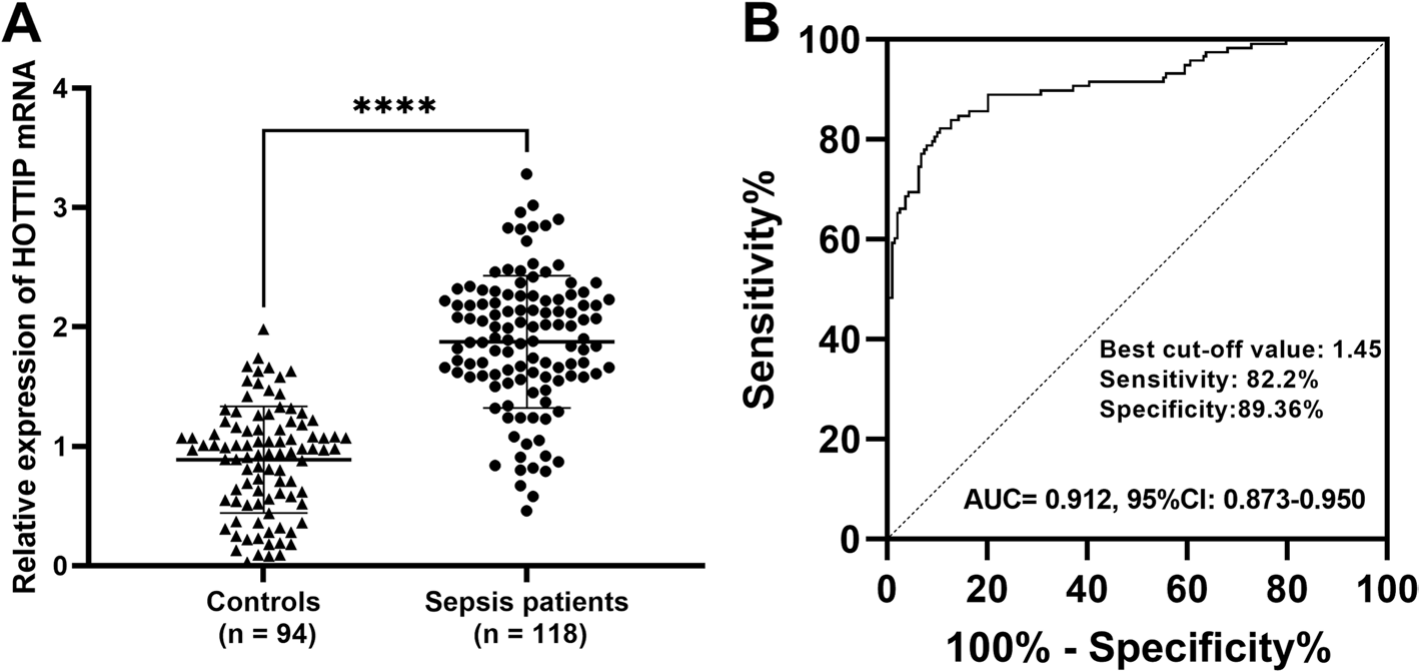
Elevated HOTTIP is a diagnostic biomarker for sepsis. **A** RT-qPCR was conducted to explore the levels of serum HOTTIP. **B** The potential diagnostic power of serum HOTTIP in patients with sepsis by ROC analysis. ****P < 0.0001 vs. controls

Serum HOTTIP was noticeably elevated in patients with sepsis compared to controls (*P* < 0.001, Fig. 1A). The AUC of the ROC curve was 0.912 (95CI%: 0.873–0.950), with a sensitivity and specificity of 82.2% and 89.36%, respectively, to identify patients with sepsis from controls when the cutoff value was 1.45 (Fig. 1B).

### Comparing clinical baseline characteristics of sepsis patients with ARDS and non-ARDS

During the 28-day follow-up, 35 (29.66%) patients with sepsis presented with ARDS. Compared to baseline information from patients with non-ARDS sepsis, patients with ARDS sepsis had a higher prevalence of smoking (*P* = 0.037), COPD disease (*P* = 0.005), and respiratory infections (*P* = 0.002), and elevated levels of CRP (*P* < 0.001) and PCT (*P* = 0.006). In terms of disease severity, patients with ARDS sepsis had higher APACHE II scores (*P* = 0.029) and SOFA scores (*P* = 0.002, Table 2) than patients with non-ARDS sepsis.

**Table 2:** Comparison of characteristics between non-ARSD sepsis patients and ARDS sepsis patients

Parameters	Non-ARDS sepsis patients (*n* = 83)	ARDS sepsis patients (*n* = 35)	*P* value
**Demographics**			
Age (year), mean ± SD	53.69 ± 10.92	56.20 ± 7.51	0.217
Gender, n (%)			
Female	36 (43.37)	16 (45.71)	0.841
Male	47 (56.63)	19 (54.29)	
BMI (kg/m^2^), mean ± SD	25.07 ± 2.42	26.07 ± 3.29	0.069
Smoke, n (%)	25 (30.12)	18 (51.43)	**0.037**
**Complications**			
COPD, n (%)	11 (13.25)	13 (37.14)	**0.005**
Cardiomyopathy, n (%)	35 (49.40)	16 (45.71)	0.841
Chronic kidney failure, n (%)	11 (13.25)	8 (22.86)	0.272
Cirrhosis, n (%)	22 (26.51)	5 (14.29)	0.230
**Primary infection site**			
Abdominal infection, n (%)	33 (39.76)	8 (22.86)	0.093
Respiratory infection, n (%)	12 (14.46)	15 (42.83)	**0.002**
Skin and soft tissue infection, n (%)	16 (19.28)	7 (20.00)	0.928
Bloodstream infection, n (%)	10 (12.05)	2 (5.71)	0.506
CNS infection, n (%)	4 (4.82)	2 (5.71)	0.840
Other infection, n (%)	8 (9.64)	1 (2.86)	0.277
**Primary organism**			
G^−^, n (%)	45 (54.22)	20 (57.14)	0.841
G^+^, n (%)	17 (20.48)	9 (25.71)	0.628
Anaerobes, n (%)	8 (9.64)	5 (14.29)	0.524
Fungus, n (%)	5 (6.02)	2 (5.71)	0.948
Mycoplasmas, n (%)	4 (4.82)	1 (2.86)	0.629
Total culture negative, n (%)	17 (20.48)	6 (17.14)	0.676
**Biochemical indexes**			
Scr (mg/dL), median (IQR)	1.9 (1.40, 2.7)	1.7 (1.4, 2.7)	0.641
Albumin, (g/L), median (IQR)	27.2 (24.3, 31.0)	25.7 (23.8, 30.7)	0.284
WBC (×10^9^/L), median (IQR)	16.5 (11.6, 27.2)	21.3 (14.6, 28.2)	0.060
CRP (mg/L), median (IQR)	83.65 (58.80, 125.06)	165.07 (77.08, 255.97)	**0.000**
PCT (ng/mL), median (IQR)	12.9 (8.9, 19.8)	18.8 (9.9, 28.7)	**0.006**
Inflammatory cytokines			
IL-1β (pg/mL)	13.5 (7.6, 19.35)	13.9 (10.9, 22.8)	0.071
IL-6 (pg/mL)	66.3 (34.7, 104.9)	78.4 (49.8, 162.1)	0.065
TNF-α (pg/mL)	181.5 (142.8, 228.4)	202.9 (157.0, 240.7)	0.063
**Disease severity**			
APAHCE II score, median (IQR)	12.0 (8.0, 16.0)	15.0 (10.0, 18.0)	**0.029**
SOFA score, median (IQR)	5.0 (4.0, 8.0)	7.0 (6.0, 10.0)	**0.002**

*BMI* Body mass index, *COPD* Chronic obstructive pulmonary disease, *CNS* Central nervous system, *APACHE* Acute physiology and chronic health evaluation, *SOFA* Sequential organ failure assessment, *Scr* Serum creatinine, *WBC* White blood cells, *CRP* C-reactive protein, *PCT* Procalcitonin, *TNF-α* Tumor necrosis factor-α, *IL* Interleukin

### Serum HOTTIP predicts the development of ARDS in sepsis patients

**Fig. 2:**
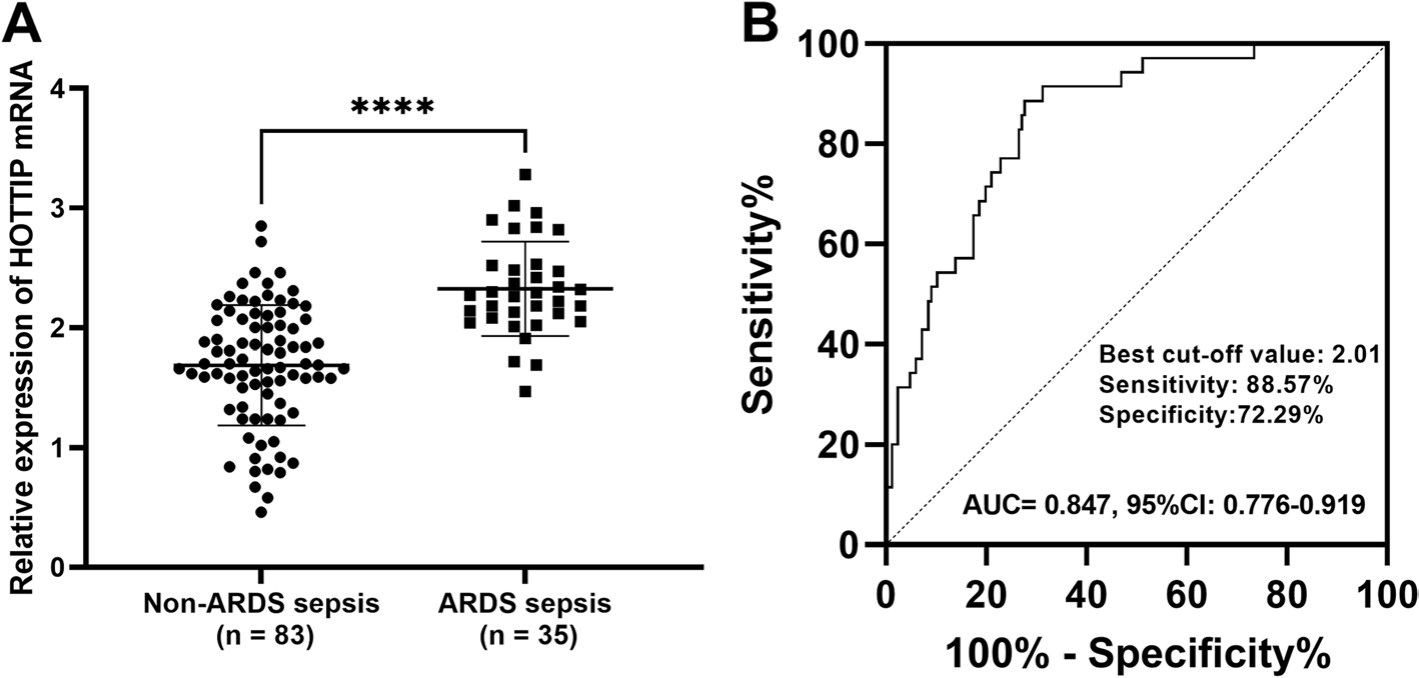
HOTTIP levels as a diagnostic biomarker for the development of ARDS in patients with sepsis. **A** The levels of HOTTIP were examined by the RT-qPCR in the patients with ARDS sepsis and non-ARDS sepsis. **B** ROC curves were plotted to explore the diagnostic value of HOTTIP in ARDS sepsis patients. ****P < 0.0001 vs. Non-ARDS sepsis groups

As illustrated in Fig. 2A, serum HOTTIP was generally higher in patients with ARDS than in patients without ARDS (*P* < 0.001). The clinical significance of HOTTIP in the development of ARDS in patients with sepsis was analyzed by plotting ROC curves according to HOTTIP levels in two groups. At a cut-off value of 2.01, serum HOTTIP clearly identified ARDS sepsis patients with an AUC of 0.847 (95%CI: 0.776–0.919), sensitivity and specificity of 88.57% and 72.29% (Fig. 2B), respectively.

Subsequently, clinical parameters and HOTTIP levels in patients with ARDS and without ARDS were incorporated as logistic variables to assess their independent effects on the occurrence of ARDS. HOTTIP levels (*P* < 0.001) can independently influence the development of ARDS, as well as COPD (*P* = 0.014), respiratory infection (*P* = 0.048), and higher CRP (*P* = 0.029, Table 3).

**Table 3:** Analysis of factors predicting ARDS in sepsis patients

Parameters	Univariate logistic regression			Multivariate logistic regression		
	HR	95%CI	*P*	HR	95%CI	*P*
Higher LnRNA HOTTIP	19.862	5.597–70.482	**< 0.001**	0.019	0.03–0.135	**< 0.001**
Higher Age	0.427	0.142–1.286	0.130	-		
Gender	1.099	0.497–2.433	0.815			
BMI (kg/m^2^)	0.746	0.338–1.649	0.469			
Smoke	0.407	0.181–0.917	**0.030**	0.339	0.082–1.390	0.133
COPD	0.259	0.102–0.658	**0.005**	0.186	0.035–0.983	**0.048**
Cardiomyopathy	1.159	0.525–2.560	0.715			
Chronic kidney failure	0.516	0.187–1.419	0.200			
Cirrhosis	2.164	0.746–6.276	0.155			
Abdominal infection	2.227	0.903–5.496	0.082			
Respiratory infection	0.225	0.091–0.558	**0.001**	0.151	0.033–0.686	**0.014**
Skin and soft tissue infection	0.955	0.354–2.575	0.928			
Bloodstream infection	2.260	0.469–10.896	0.310			
CNS infection	0.835	0.146–4.785	0.840			
Other infection	0.276	0.033–2.293	0.233			
G^−^	0.888	0.400–1.970	0.770			
G^+^	0.744	0.295–1.880	0.532			
Anaerobes	0.640	0.194–2.114	0.464			
Fungus	1.058	0.195–5.730	0.948			
Mycoplasmas	1.722	0.186–15.976	0.633			
Total culture negative	1.245	0.445–3.480	0.676			
Higher Scr	1.629	0.735–3.606	0.229			
Higher Albumin	1.330	0.596–2.965	0.486			
Higher WBC	0.536	0.240–1.197	0.128			
Higher CRP	0.334	0.145–0.771	**0.010**	0.220	0.057–0.853	**0.029**
Higher PCT	0.334	0.145–0.771	**0.010**	0.664	0.158–2.788	0.576
Higher IL-1β	0.338	0.144–0.791	**0.012**	0.260	0.056–1.201	0.084
Higher IL-6	0.563	0.252–1.256	0.160			
Higher TNF-α	0.475	0.211–1.069	0.072			
Higher APAHCE II score	2.961	1.264–6.934	**0.012**	0.364	0.095–1.404	0.142
Higher SOFA score	5.453	2.049–14.515	**0.001**	2.808	0.737–10.700	0.130

*BMI* Body mass index, *COPD* Chronic obstructive pulmonary disease, *CNS* Central nervous system, *APACHE* Acute physiology and chronic health evaluation, *SOFA* Sequential organ failure assessment, *Scr* Serum creatinine, *WBC* White blood cells, *CRP* C-reactive protein, *PCT* Procalcitonin, *TNF-α* Tumor necrosis factor-α, *IL* Interleukin

### Correlation of HOTTIP levels with disease severity in patients with sepsis

**Fig. 3:**
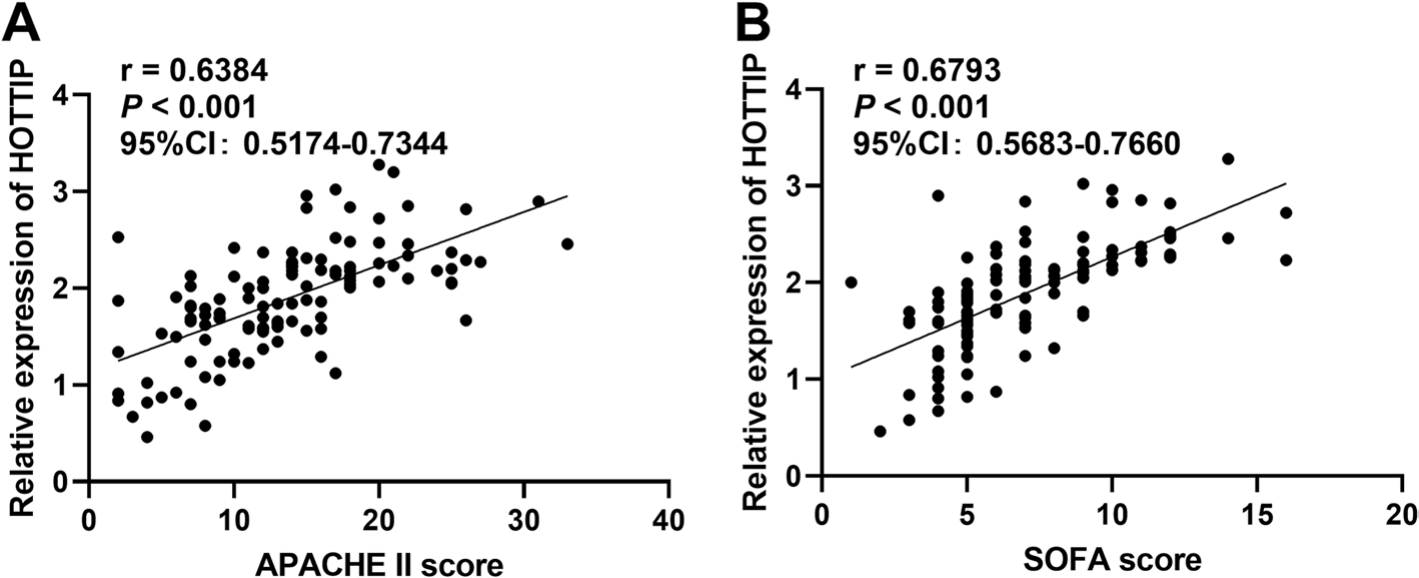
The Spearman coefficient confirmed that APAHCE II score (**A**) and SOFA score (**B**), the indicator of sepsis severity, were positively correlated with serum HOTTIP, respectively

Spearman coefficient analysis revealed a positive correlation between serum HOTTIP levels and APACHE II scores in patients with sepsis (*r* = 0.6384, Fig. 3A). Meanwhile, serum HOTTIP levels were also associated with the SOFA score (*r* = 0.6793, Fig. 3B). The results suggest that the serum HOTTIP level is correlated with the severity of sepsis.

### HOTTIP levels can predict 28-day death in patients with sepsis

**Fig. 4:**
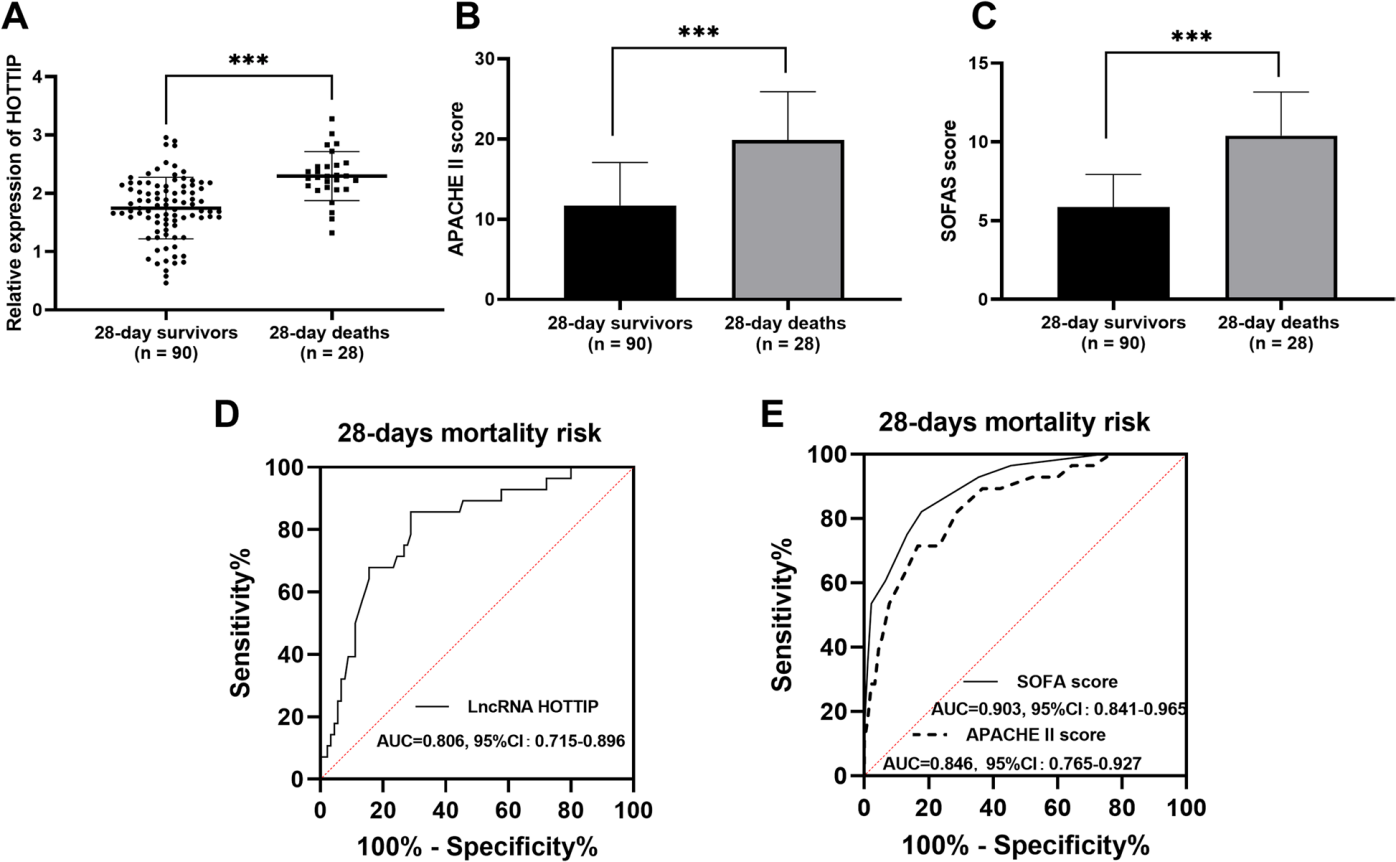
Serum HOTTIP significantly predicts 28-day mortality in patients with sepsis. Serum HOTTIP levels (**A**), APACHE II score (**B**), and SOFA score (**C**) in sepsis survivals during the 28-day follow-up were analyzed. ROC curve examines the predictive value of HOTTIP (**D**), SOFA scores, and APACHE II scores (**E**) on patient mortality. ***P < 0.0001 vs. 28-day survivors

During the 28-day follow-up period, 28 (23.73%) patients with sepsis experienced death. The serum HOTTIP was generally elevated in deaths than in survivors (*P* < 0.001, Fig. 4A). Furthermore, the patients who died of sepsis exhibited significantly elevated APACHE II scores and SOFA scores compared to survivors (*P* < 0.001, Fig. 4B-C). The ROC curves indicate that HOTTIP levels have predictive power for mortality in patients with sepsis, with an AUC of 0.806, sensitivity and specificity of 85.71% and 71.11%, respectively, at a cutoff value of 2.045 (Fig. 4D). This is similar to the ability of the APACHE II score and the SOFA score to predict sepsis mortality, with an AUC of 0.903 and 0.846, respectively (Fig. 4E).

**Fig. 5:**
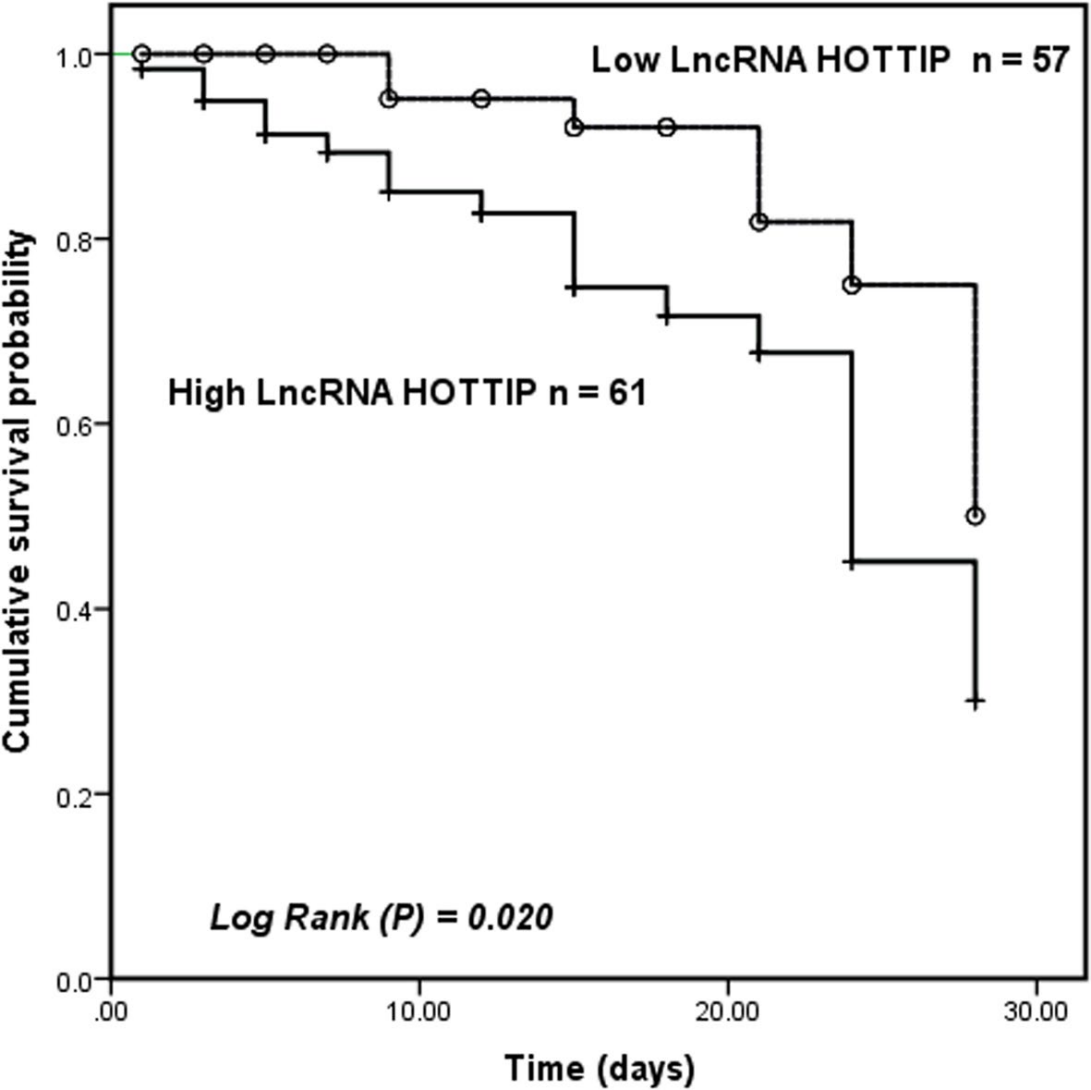
Kaplan-Meier analysis to check the contribution of HOTTIP levels on the progression of patients with sepsis

Finally, patients with sepsis were grouped according to mean HOTTIP levels (1.88 ± 0.55), and Kaplan-Meier curves were plotted. As illustrated in Fig. 5, mortality was higher in patients with high levels of HOTTIP (log Rank *P* = 0.020), and serum HOTTIP (HR = 4.813, 95%CI: 1.471–15.750, *P* = 0.009, Table 4) was an independent predictor of death in patients with sepsis.

**Table 4:** Cox regression analysis of independent risk factors for 28-day mortality in patients with sepsis

Parameters	Univariate COX			Multivariate COX		
	HR	95%CI	*P*	HR	95%CI	*P*
LnRNA HOTTIP	2.46	1.08–5.58	**0.032**	4.813	1.471–15.750	**0.009**
Age	0.847	0.402–1.78	0.661			
Gender	1.318	0.557–2.55	0.705			
BMI (kg/m^2^)	0.967	0.242–3.867	0.962			
Smoke	1.652	0.753–3.624	0.211			
COPD	2.361	1.112–5.011	**0.025**	1.770	0.745–4.208	0.196
Cardiomyopathy	0.570	0.266–1.224	0.149			
Chronic kidney failure	0.479	0.203–1.131	0.093			
Cirrhosis	0.946	0.356–2.510	0.911			
Abdominal infection	0.721	0.337–1.544	0.400			
Respiratory infection	2.220	1.053–4.681	**0.036**	1.666	0.657–4.227	0.282
Skin and soft tissue infection	1.115	0.451–2.757	0.814			
Bloodstream infection	0.413	0.056–3.044	0.386			
CNS infection	0.334	0.077–1.450				
Other infection	0.924	0.122–7.017	0.939			
G^−^	0.994	0.472–2.091	0.987			
G^+^	0.910	0.369–2.245	0.837			
Anaerobes	0.462	0.109–1.963	0.462			
Fungus	0.428	0.148–1.237	0.117			
Mycoplasmas	0.694	0.162–2.970	0.623			
Total culture negative	0.921	0.350–2.423	0.867			
Scr	2.361	1.038–5.373	**0.041**	1.392	0.567–3.415	0.470
Albumin	2.014	0.497–2.225	0.072			
WBC	2.073	0.936–4.589	0.072			
CRP	2.371	1.083–5.189	**0.031**	1.682	0.636–4.452	0.295
PCT	2.285	1.006–5.189	**0.048**	2.046	0.633–6.611	0.232
IL-1β	2.368	1.100-5.096	**0.028**	1.896	0.777–4.624	0.160
IL-6	2.231	1.034–4.810	**0.041**	2.012	0.805–5.029	0.135
TNF-α	2.346	1.058–5.203	**0.036**	1.738	0.696–4.342	0.237
APAHCE II score	2.581	1.188–5.610	**0.017**	2.920	1.050–8.117	**0.040**
SOFA score	2.265	1.063–4.826	**0.034**	2.790	1.094–7.118	**0.032**

*BMI* Body mass index, *COPD* Chronic obstructive pulmonary disease, *CNS* Central nervous system, *APACHE* Acute physiology and chronic health evaluation, *SOFA* Sequential organ failure assessment, *Scr* Serum creatinine, *WBC* White blood cells, *CRP* C-reactive protein, *PCT* Procalcitonin, *TNF-α* Tumor necrosis factor-α, *IL* Interleukin

### HOTTIP functions as a sponge of miR-574-5p

**Fig. 6:**
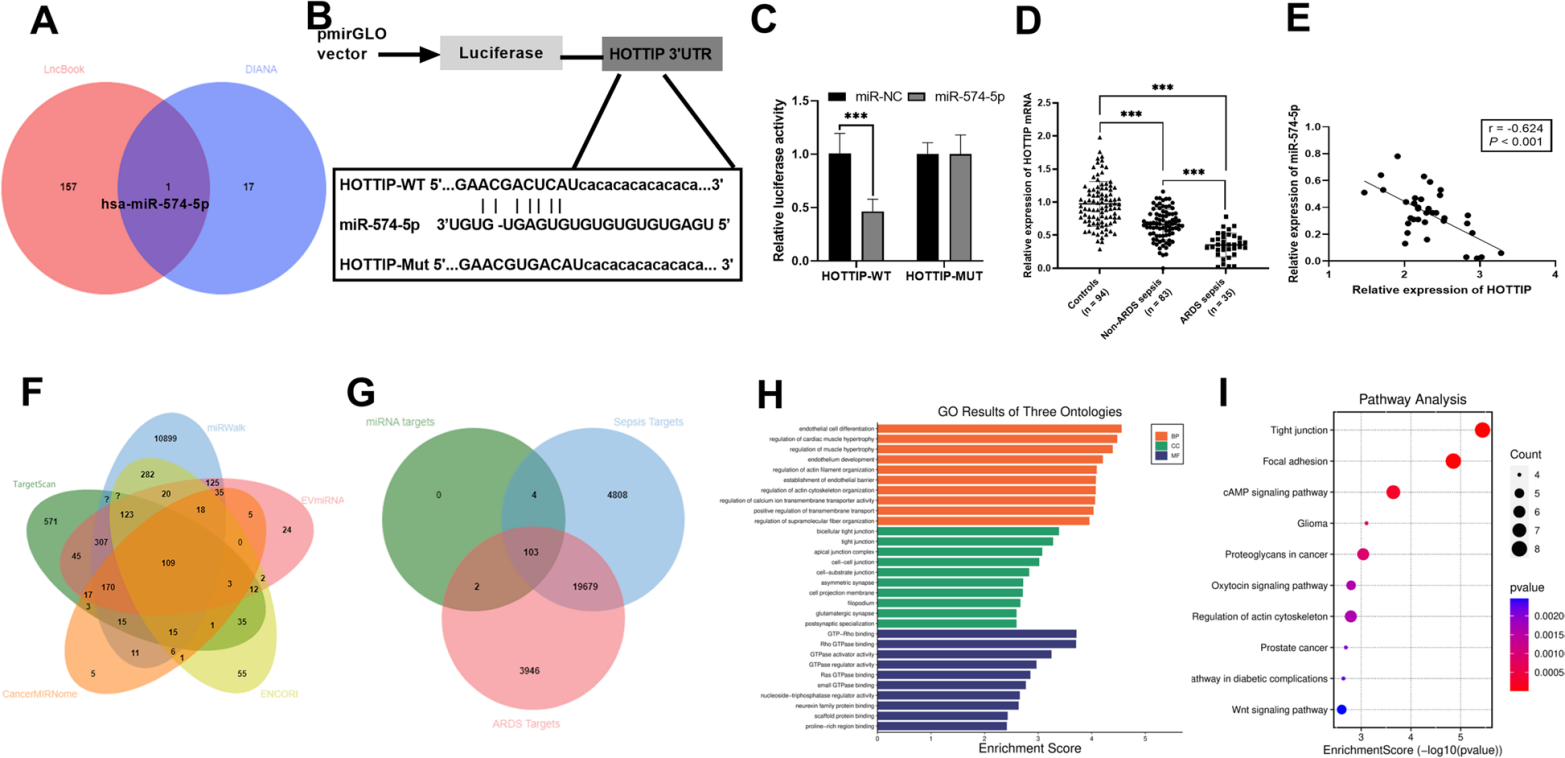
Targets genes of HOTTIP and bioinformatics analysis. **A** Veen diagram presenting target miRNAs predicted for HOTTIP in LncBook and DIANA databases. **B** Targeted binding site between HOTTIP and miR-574-5p. **C** Dual-luciferase reporter gene validates the targeting relationship between miR-574-5p and HOTTIP. **D** Serum miR-574-5p levels were assessed by the RT-qPCR. **E** Pearson’s correlation analysis was employed to examine the relationship between serum miR-574-5p levels and HOTTIP levels in patients with ARDS sepsis. **F** Veen diagram presenting multiple databases predicting overlapping target genes for miR-574-5p. **G** Veen’s diagram presented the overlapping genes between miR-574-5p targets genes and sepsis, ARDS-related genes, respectively. The biological function and pathway of the overlapping genes were identified through the bioinformatics platform, which makes use of GO (**H**) and KEGG (**I**) pathway (guidelines: www.kegg.jp/kegg/kegg1.html). ***P < 0.0001 vs. miR-NC, controls, or Non-ARDS sepsis

As shown in Fig. 6A, the overlapping target miRNA for HOTTIP in the LncBook and DIANA databases was miR-574-5p. Figure 6B shows the potential binding sites of miR-574-5p with HOTTIP. Compared to miR-NC, miR-574-5p mimic markedly reduced the luciferase activity of HOTTIP-WT but had no meaningful effect on the luciferase activity of HOTTIP-MUT (*P* < 0.05, Fig. 6C). Furthermore, serum miR-574-5p was noticeably lower in patients with sepsis compared to controls (*P* < 0.05), and the lowest levels were found in patients with ARDS sepsis (*P* < 0.05, Fig. 6D). Serum miR-574-5p levels were negatively correlated with HOTTIP levels in patients with ARDS sepsis (*P* < 0.001, *r* = -0.624, Fig. 6E).

### GO enrichment and KEGG enrichment of mir-574-5p target genes

Finally, the potential mechanisms underlying the action of miR-574-5p were investigated. Potential targets for miR-574-5p were predicted by 5 databases and 109 overlapping genes were identified (Fig. 6F and Supplementary Tabel 1). The overlapping gene was then again subjected to Venn diagram analysis with sepsis target genes and ARDS target genes pooled in the CTD database to look for genes associated with ARDS sepsis, and 103 overlapping genes were identified (Fig. 6G, Supplementary Table 2). GO enrichment analysis revealed that the biological processes (BP) of the target genes were primarily enriched in endothelial cell differentiation, the cell component class (CC) was enriched in the tight junction of the bicellular region, and the molecular functions (MF) were primarily enriched in the binding of GTP-Rho (Fig. 6H, and Supplementary Tabel 3). KEGG enrichment analysis of the signaling pathway of overlapping target genes, a total of 190 signaling pathways were enriched, such as tight junction, focal adhesion, and the Wnt signaling pathway (Fig. 6I and Supplementary Tabel 4).

## Discussion

Significant advances have been achieved in understanding and managing ARDS to data, but the prevalence of ARDS remains elevated, particularly among patients with sepsis [5]. Currently, the diagnoses of ARDS are mostly formulated clinical features, and the commonly used clinical criteria are the Berlin definition or the American European Consensus Conference (AECC) criteria. Recently, the application of biomarkers in ARDS gained attention from both researchers and clinical practitioners. In contrast, clinical diagnosis is more inclined to clinical manifestations, with some subjective influence. However, biomarkers are objectively measured, without bias in personal understanding, and are more advantageous in the future diagnosis and treatment of ARDS.

Increasing evidence suggests that LncRNAs can be used as potential diagnostic and prognostic biomarkers of ARDS in sepsis, such as MALAT1 [19] and PVT1 [20]. The early stage of ARDS is characterized by inflammation. Previous studies identified HOTTIP as inflammatory regulators involved in multiple disease progression. For example, HOTTIP participates in acute gouty arthritis through miR-101-3p/BRD4 [7] and is associated with rheumatoid arthritis [21]. The suppression of HOTTIP also typically alleviated neuroinflammatory damage in Parkinson’s [22] and high glucose-induced inflammation in diabetic nephropathy [9]. Elevated HOTTIP has been demonstrated in atherosclerosis and its levels are induced by pro-inflammatory TNF-α [23]. Additionally, dysregulated HOTTIP is involved in oral mucosal inflammation [24], hepatitis B [25], and inflammation in systemic sclerotic disease [26]. Furthermore, dysregulated HOTTIP has been reported in respiratory diseases. HOTTIP is up-regulated in asthmatic mice and is associated with inflammation factor secretion [8]. It is also associated with the prognosis of patients with small lung cancer [27]. Furthermore, ARDS is caused by a variety of endogenous and exogenous injury factors the result in severe damage to the alveolar-capillary barrier, producing interstitial and alveolar edema, as well as the formation of hyaline membranes on the alveolar surface. This pathological process progresses progressively to pulmonary fibrosis. And pulmonary fibrosis is a major cause of poor prognosis in ARDS in patients with sepsis [2]. Importantly, HOTTIP was noticeably upregulated in patients with pulmonary fibrosis and enhanced lung tissue fibrosis via miR-744-5p/PTBP1 [10].

In particular, Fan et al. found that HOTTIP was significantly elevated in patients with sepsis patients and was associated with cardiac dysfunction [28]. Consistently, HOTTIP was also found to be generally elevated in sepsis patients in our investigation. Elevated HOTTIP may be a potential diagnostic biomarker for sepsis. However, in this study, it was found for the first time that HOTTIP was usually higher in patients with ARDS sepsis than those without ARDS. The findings suggest that dysregulated HOTTIP may be involved in the progression of ARDS in sepsis. To confirm our suspicions, we included clinical information and HOTTIP levels in a logistic regression analysis and found that HOTTIP and COPD, respiratory infection, and CRP were all independent predictors of the risk of ARDS in patients with sepsis. CRP, as an indicator of inflammation, is also abnormally expressed in medicine infections (urology, sepsis, meningitis, osteomyelitis) and surgical disorders (acute appendicitis, tissue injuries, postoperative infections), cardiovascular disorders, systemic infectious diseases, as well as malignant disorders, and even diseases related to intensive care units, and lack some specificity [29]. Furthermore, CRP requires multiple measurements at 6–12 h intervals to have a greater diagnostic value. However, HOTTIP, as an ncRNA, has some stability that can be detected only at the time of admission and is only abnormally expressed in some parts of the disease with high specificity. At the same time, HOTTIP levels have some precision in diagnosing sepsis patients who develop ARDS from sepsis patients and have the potential as a diagnostic biomarker for sepsis patients with ARDS.

Furthermore, we study the correlation between HOTTIP and the severity of sepsis. The SOFA score and the APACHE II score are widely used in clinical practice as commonly used scoring tools to assess the severity of sepsis, in the belief that they are independently associated with in-hospital mortality in patients with sepsis [30]. In our study, HOTTIP levels were found to be positively related to the SOFA score and the APACHE II score, respectively, suggesting that HOTTIP may have an impact on the condition or death of a patient with sepsis. Previous studies have highlighted HOTTIP as a biomarker for NSCLC recurrence [31] and as a prognostic biomarker for patients with gastric cancer [32]. Although the impact of HOTTIP levels on the prognosis of patients with sepsis is currently unknown, to fill this gap, we conducted a short-term follow-up study of included patients over 28 days. During the follow-up period, a total of 28 patients death. Further analysis revealed generally higher levels of HOTTIP in deaths than in survivors. Furthermore, elevated HOTTIP, as well as APACHE II and SOFA scores [33, 34], predicted the appearance of death in patients with sepsis and were both independent predictors of death in the patients with sepsis. However, HOTTIP has its unique advantages over the two scores in terms of predictive value, firstly given that HOTTIP, as an ncRNA, may already be resentful in early changes in the disease and its prediction may be earlier than the two scores. Furthermore, the APACHE II and SOFA scores are derived based on the range of clinical indicators, while the detection of HOTTIP levels is simple in comparison. However, larger and more clinical samples are needed for further validation. Based on the above studies and our experimental results, we postulate that HOTTIP is may be involved in the progression of sepsis by modulating the inflammatory response and pulmonary fibrosis, which will be addressed in our further studies.

Mechanistically, LncRNAs are molecular sponges for miRNAs that adsorb them, thereby repressing miRNA expression. In this study, we tried to explore target miRNAs for the HOTTIP action, and the only overlapping target miRNA predicted by the database was miR-574-5p. miR-574-5p is located on human chromosome 4p14. It was identified as sepsis-associated miRNA, and downregulation of its levels can predict the development of acute kidney injury in patients with sepsis [35]. MiR-574-5p was significantly reduced in acute lung injury associated with sepsis [36] and was recognized as a miRNA associated with death in patients with sepsis [37]. Consistent with previous studies, we also found that miR-574-5p levels were generally lower in patients with sepsis. And ARDS patients with sepsis had lower levels of miR-574-5p. We then predicted the target genes of miRNA action and analyzed the overlap with genes related to ARDS and sepsis, identifying 103 overlapping genes. GO comments revealed that these overlapping genes are primarily enriched in endothelial-related biological processes, such as endothelial cell differentiation, endothelium development, and establishment of the endothelial barrier. Previous studies have reported that endothelial cell barrier dysfunction attenuates acute lung injury induced by sepsis [38], and that endothelial dysfunction is also involved in the pathogenesis and prognosis of sepsis ARDS [39, 40]. Additionally, both tight junction and focal adhesion are key components of cellular connectivity, and the integrity of endothelial cell adhesion links is involved in lung injury in sepsis [41]. Unseparated heparin attenuates sepsis-induced acute lung injury by protecting tight junctions [42]. KEGG enrichment of overlapping target genes for tight junction and focal adhesion. Here, we suspect that HOTTIP may be involved in the progression of ARDS sepsis by acting on miR-574-5p and endothelial-related target genes that modulate signaling pathways, such as cell junctions. Additionally, the WNT signaling pathway was also enriched in KEGG. Previous studies have found that lung repair and regeneration in ARDS are associated with WNT signaling [43] and that the WNT signaling pathway is involved in the trans differentiation of alveolar epithelial type II cells and promotes the progression of ARDS [44].

However, the specific molecular mechanism of the HOTTIP/miR-574-5p axis will be covered in the next studies. There are undeniable limitations to this study. First, this case-control study was conducted in a single center and the sample size was compelled by the available number of patients with sepsis. Second, APACHE III scores have recently been found to show higher differentiation in hospital mortality. However, we have used the earlier and more widely available APACHE II scores, this is a potential limitation of this preliminary study. The APACHE III score will be used for validation in subsequent multicenter in-depth studies with larger sample sizes. Furthermore, although we selected 103 overlapping genes, it is necessary to further investigate the mRNAs that act on the HOTTIP/miR-574-5p axis, which may be a key target for the treatment of ARDS in sepsis. Finally, the detection of HOTTIP levels in clinical practice may need to be performed in well-equipped hospitals, so its generalizability in clinical practice needs to be further explored. Nevertheless, our study broadens new perspectives for the diagnosis of sepsis, but further exploration of biomarkers that simplify the testing procedure and are cost-effective for clinical practice is needed.

## Conclusions

In conclusion, elevated HOTTIP is a potential diagnostic biomarker of sepsis ARDS and can predict the onset of short-term mortality. And this may be achieved by the miR-574-5p sponge. Our study expands new ideas for the management of sepsis ARDS.

### Supplementary Information

**Additional file 1.** STROBE Statement—checklist of items that should be included in reports of observational studies.

**Additional file 2: Supplementary Figure.** The flow chart diagram of patient inclusion. **Supplementary Table 1.** Identified 109 overlapping target genes of miR-574-5p in 5 databases. **Supplementary Tabel 2.** Overlapping genes between miR-574-5p predicted genes and sepsis, ARDS-related genes. **Supplementary Table 3.** Top 20 significant GO terms of the overlapped genes. **Supplementary Table 4. **Top 10 significant enriched pathways of the overlapped genes.

## Data Availability

The datasets used and/or analyzed during the current study are available from the corresponding author upon reasonable request.

## Abbreviations

ARDS: Acute respiratory distress syndrome
BMI: Body mass index
GO: Gene Ontology
HOTTIP: HOXA distal transcript antisense RNA
KEGG: Kyoto Encyclopedia of Genes and Genomes
LncRNAs: Long noncoding RNAs
MUT: Mutant
ROC: Receiver operating C characteristic
RT-qPCR: Real-time polymerase chain reaction
WT: Wild-type
