# Supplementary material for: LncRNA HOTTIP as a diagnostic biomarker for acute respiratory distress syndrome in patients with sepsis and to predict the short-term clinical outcome: a case-control study
Source: BMC Anesthesiol. 2024 Jan 18;24:30. doi: 10.1186/s12871-024-02405-z (PMC10795278; doi:10.1186/s12871-024-02405-z)
Supplement: Supplementary file 1 — Additional file 1. STROBE Statement—checklist of items that should be included in reports of observational studies. [file 12871_2024_2405_MOESM1_ESM.docx]

STROBE Statement—checklist of items that should be included in reports of observational studies

|  | Item No. | Recommendation | Page  No. | Relevant text from manuscript |
| --- | --- | --- | --- | --- |
| **Title and abstract** | 1 | (*a*) Indicate the study’s design with a commonly used term in the title or the abstract | 1 | LncRNA HOTTIP as a diagnostic biomarker for acute respiratory distress syndrome in patients with sepsis and to predict short-term clinical outcome: A case-control study |
|  |  | (*b*) Provide in the abstract an informative and balanced summary of what was done and what was found | 2 | Current study revealed that HOTTIP was a diagnostic biomarker for the development of ARDS, correlated with the severity of the disease, and predicted short-term death in sepsis patients. HOTTIP may be involved in ARDS progression by targeting miR-574-5p. |
| Introduction | | | |  |
| Background/rationale | 2 | Explain the scientific background and rationale for the investigation being reported | 3 | Dysregulation of long noncoding RNAs (LncRNAs) was identified as the culprit in the pathogenesis of ARDS in sepsis. Moreover, elevated HOTTP was also observed in patients with sepsis and was associated with their cardiac dysfunction. However, the potential function of HOTTIP in sepsis-induced ARDS remains unclear. |
| Objectives | 3 | State specific objectives, including any prespecified hypotheses | 4 | Based on the above information, we hypothesize that HOTTIP plays a crucial function in the ARDS of sepsis, and we focus on its diagnostic and prognostic significance for ARDS patients. |
| Methods | | | |  |
| Study design | 4 | Present key elements of study design early in the paper | 4 | This study was a case-control study of unmatched design. Therefore, a minimum sample size of 94 participants per group was required, and we included a strict minimum sample size in this unmatched design case-control study in the control group but included more than the minimum number of patients in the patient group to the best of our ability. |
| Setting | 5 | Describe the setting, locations, and relevant dates, including periods of recruitment, exposure, follow-up, and data collection | 4-5 | 118 patients with sepsis admitted to The First People's Hospital of Xuzhou from January 2019 to June 2020 were included. Record demographic characteristics of subjects, including age, gender, and body mass index (BMI) after admission. Biological indicators such as scr, WBC, and CRP were also analysed. APACHE II score and SOFA score, indicators of the severity of sepsis and severity of organ failure impairment, were assessed within 24 h of admission. Blood was collected from subjects on the day of admission, while controls were collected at enrolment. The blood samples were centrifuged, and the upper serum was stored at -80℃  Following admission, patients were given standard treatment and resuscitated. The experiment started with the patient’s diagnosis of sepsis and ended with short-term monitoring of the patient over 28 days, recording the number of days from admission to death or last visit. The cumulative survival probability was calculated, and the predictive power of HOTTIP for clinical outcomes was examined. |
| Participants | 6 | (*a*) *Cohort study*—Give the eligibility criteria, and the sources and methods of selection of participants. Describe methods of follow-up  *Case-control study*—Give the eligibility criteria, and the sources and methods of case ascertainment and control selection. Give the rationale for the choice of cases and controls  *Cross-sectional study*—Give the eligibility criteria, and the sources and methods of selection of participants | 4 | This study was a case-control study of unmatched design. And the trial was conducted in accordance with the STROBE Statement checklist of items that should be included in reports of case-control studies |
|  |  | (*b*) *Cohort study*—For matched studies, give matching criteria and number of exposed and unexposed  *Case-control study*—For matched studies, give matching criteria and the number of controls per case | 4 | What’s more, a control group of 94 healthy volunteers from the physical examination canter was enrolled. They were matched for age and gender to patients with sepsis and had no recent immunosuppressive medication, no history of sepsis, and all had normal physical examination parameters. |
| Variables | 7 | Clearly define all outcomes, exposures, predictors, potential confounders, and effect modifiers. Give diagnostic criteria, if applicable | 4-5 | diagnosis as sepsis under the 3rd International consensus definitions of sepsis. Monitored for the onset of ARDS promptly and assessed based on the Berlin definition (11), which included: a) acute onset with novel or aggravated respiratory symptoms within 1 week; b) chest imaging shows diffuse infiltrative shadows in both lungs that cannot be fully interpreted as exudates, atelectasis, masses; c) The origin of edema and respiratory failure cannot be explained by heart failure or excessive fluid input. |
| Data sources/ measurement | 8* | For each variable of interest, give sources of data and details of methods of assessment (measurement). Describe comparability of assessment methods if there is more than one group | 4-5 | Record demographic characteristics of subjects, including age, gender, and body mass index (BMI) after admission. Biological indicators such as scr, WBC, and CRP were also analyzed. APACHE II score and SOFA score, indicators of the severity of sepsis and severity of organ failure impairment, were assessed within 24 h of admission. Blood was collected from subjects on the day of admission, while controls were collected at enrollment. The blood samples were centrifuged, and the upper serum was stored at -80℃.  The Easypure miRNA kit was utilized to isolate total serum RNA and examine its quality on a NanoDrop 1000 spectrophotometer. 2 μg RNA was synthesized into cDNA by TranscScript Two-Step RT-PCR superMix kit on a PCR amplifier. Then, SYBR Green qPCR Master Mix Kit reagent, cDNA, primers, and RNase Free H2O were added to the EP tubes and mixed to perform RT-qPCR amplification reactions in the CFX96 real-time PCR system. GAPDH served as an internal control for LncRNA, quantified by the 2−ΔΔCt method and calculated after three independent replicate experiments. |
| Bias | 9 | Describe any efforts to address potential sources of bias | 12 | There are undeniable limitations to this study. Firstly, this case-control study was conducted at a single centre and the sample size was obliged by the available number of patients with sepsis. Secondly, APACHE III scores have recently been found to show higher differentiation in in-hospital mortality. However, we have used the earlier and more widely available APACHE II scores, this is a potential limitation of this preliminary study. The APACHE III score will be used for validation in subsequent multicentre in-depth studies with larger sample sizes. Finally, although we screened out 103 overlapping genes, there is a need to further delve into the mRNAs that act on the HOTTIP/miR-574-5p axis, which may be a key target for the treatment of ARDS in sepsis. |
| Study size | 10 | Explain how the study size was arrived at | 4 | The prevalence of sepsis with ARDS is about 25% (2,11), while the prevalence of ARDS alone is about 9.5% (12). According to the sample size calculation formula of the case-control study with an unmatched design (13,14): n=2×(p (1-p) (U_α_+U_β_ )^2)/(p_1_-p_2_)^2 , a two-sided test was used with values of α = 0.05 and β=0.20, Uα = 1.96 (from Z table) at type I error of 5%，U_β_= 0.84 (from Z table) at 80% power, p1 = prevalence in case group (p1 = 25), p2 = prevalence in control group (p2 = 9.5), p = pooled prevalence = [p1+p2]/2. Therefore, a minimum sample size of 94 participants per group was required, and we included a strict minimum sample size in this unmatched design case-control study in the control group but included more than the minimum number of patients in the patient group to the best of our ability. |

Continued on next page

| Quantitative variables | 11 | Explain how quantitative variables were handled in the analyses. If applicable, describe which groupings were chosen and why | 6 | RT-qPCR was used to quantify HOTTIP expression levels in patients with sepsis (including Non-ARDS and ARDS) and controls. Data were compared between two groups using Student’s T-test and differences between multiple groups were analyse using one-way and two-way ANOVA followed by Tukey's post-hot test |
| --- | --- | --- | --- | --- |
| Statistical methods | 12 | (*a*) Describe all statistical methods, including those used to control for confounding | 6 | Data were compared between two groups using Student’s T-test and differences between multiple groups were analyse using one-way and two-way ANOVA followed by Tukey's post-hot test. Receiver operating C characteristic (ROC) was carried out to examine the diagnostic significance. Log-rank test of Kapan-Meier curves to explore the predictive significance on short-term survival outcomes. |
|  |  | (*b*) Describe any methods used to examine subgroups and interactions | 6 | Data were compared between two groups using Student’s T-test and differences between multiple groups were analyse using one-way and two-way ANOVA followed by Tukey's post-hot test. |
|  |  | (*c*) Explain how missing data were addressed | 4 | The exclusion criteria of the group were e) patients with incomplete or missing clinical data. |
|  |  | (*d*) *Cohort study*—If applicable, explain how loss to follow-up was addressed  *Case-control study*—If applicable, explain how matching of cases and controls was addressed  *Cross-sectional study*—If applicable, describe analytical methods taking account of sampling strategy | 4 | Hospital based unmatched case control study design was employed. Therefore, a minimum sample size of 94 participants per group was required, and we included a strict minimum sample size in this unmatched design case-control study in the control group but included more than the minimum number of patients in the patient group to the best of our ability. |
|  |  | (*e*) Describe any sensitivity analyses | 5 | . Receiver operating C characteristic (ROC) was carried out to examine the diagnostic significance (sensitivity and specificity). |
| Results | | | | |
| Participants | 13* | (a) Report numbers of individuals at each stage of study—eg numbers potentially eligible, examined for eligibility, confirmed eligible, included in the study, completing follow-up, and analysed | 6 | A total of 118 patients with sepsis were enrolled; their mean age was 54.43±10.07 years, of which 55.93% were male. Primary sites of infection included 41 (34.75%) abdominal infections, 27 (22.88%) respiratory infections, 23 (19.49%) skin and soft tissue infections, 12 (10.17%) bloodstream infections, 6 (5.08%) CNS infections, and 9 (7.63%) other infections. |
|  |  | (b) Give reasons for non-participation at each stage | 3 | a) patients who had received immunosuppressive therapy within the last 6 months; b) chronic organ failure; c) complicated with severe cardiac, liver, and renal impairment; d) death within 24 h of admission; e) patients with incomplete or missing clinical data. |
|  |  | (c) Consider use of a flow diagram | 4 | Supplement material Figure-flow diagram |
| Descriptive data | 14* | (a) Give characteristics of study participants (eg demographic, clinical, social) and information on exposures and potential confounders | 6 | A total of 118 patients with sepsis were enrolled; their mean age was 54.43±10.07 years, of which 55.93% were male. Primary sites of infection included 41 (34.75%) abdominal infections, 27 (22.88%) respiratory infections, 23 (19.49%) skin and soft tissue infections, 12 (10.17%) bloodstream infections, 6 (5.08%) CNS infections, and 9 (7.63%) other infections. Additionally, the median values of biochemical parameters Scr, Albumin, WBC, CRP, and PCT were 1.9 (1.4, 2.7) mg/dL, 27.2 (23.8, 30.7) g/L, 17.5 (12.6, 27.3) WBC109/L, 91.96 (60.88, 143.81) mg/L, 15.4 (8.9, 21.8) ng/mL. The inflammatory factors IL-1β, IL-6, and TNF-α were 13.8 (8.8, 20.2) pg/mL, 70.9 (45.2, 111.9) pg/mL, and 188.1 (146.6, 233.8) g/mL. Detailed information was recorded in Table 1. |
|  |  | (b) Indicate number of participants with missing data for each variable of interest | 6 | patients with incomplete or missing clinical data were excluded. |
|  |  | (c) *Cohort study*—Summarise follow-up time (eg, average and total amount) |  | NO |
| Outcome data | 15* | *Cohort study*—Report numbers of outcome events or summary measures over time |  | NO |
|  |  | *Case-control study—*Report numbers in each exposure category, or summary measures of exposure | 6 | A total of 118 patients with sepsis were enrolled; their mean age was 54.43±10.07 years, of which 55.93% were male. Primary sites of infection included 41 (34.75%) abdominal infections, 27 (22.88%) respiratory infections, 23 (19.49%) skin and soft tissue infections, 12 (10.17%) bloodstream infections, 6 (5.08%) CNS infections, and 9 (7.63%) other infections. Additionally, the median values of biochemical parameters Scr, Albumin, WBC, CRP, and PCT were 1.9 (1.4, 2.7) mg/dL, 27.2 (23.8, 30.7) g/L, 17.5 (12.6, 27.3) ×109/L, 91.96 (60.88, 143.81) mg/L, 15.4 (8.9, 21.8) ng/mL. The inflammatory factors IL-1β, IL-6, and TNF-α were 13.8 (8.8, 20.2) pg/mL, 70.9 (45.2, 111.9) pg/mL, and 188.1 (146.6, 233.8) g/mL. In addition, a control group of 94 healthy volunteers from the physical examination centre was enrolled. Detailed information was recorded in Table 1. |
|  |  | *Cross-sectional study—*Report numbers of outcome events or summary measures |  | NO |
| Main results | 16 | (*a*) Give unadjusted estimates and, if applicable, confounder-adjusted estimates and their precision (eg, 95% confidence interval). Make clear which confounders were adjusted for and why they were included | 8 | mortality was higher in patients with high levels of HOTTIP (log-Rank P = 0.020), and serum HOTTIP (HR = 4.813. 95%CI: 1.471-15.750, P = 0.009, Table 4) was an independent predictor of death in patients with sepsis. |
|  |  | (*b*) Report category boundaries when continuous variables were categorized | 8 | sepsis patients were grouped according to the mean HOTTIP levels (1.88±0.55), and Kaplan-Meier curves were plotted. |
|  |  | (*c*) If relevant, consider translating estimates of relative risk into absolute risk for a meaningful time period |  | NO |

Continued on next page

| Other analyses | 17 | Report other analyses done—eg analyses of subgroups and interactions, and sensitivity analyses | 7-8 | ROC curves indicate that HOTTIP levels have predictive power for the occurrence of death in sepsis patients with an AUC of 0.806, sensitivity and specificity of 85.71% and 71.11%, respectively, at a cut-off value of 2.045 (**Fig. 4D**). This is similar to the ability of APACHE II score and SOFA score (**Fig. 4E**) to predict sepsis mortality, with an AUC of 0.903 and 0.846, respectively. |
| --- | --- | --- | --- | --- |
| Discussion | | | | |
| Key results | 18 | Summarise key results with reference to study objectives | 12 | In conclusion, elevated HOTTIP is a potential diagnostic biomarker for sepsis ARDS and can predict the onset of short-term mortality. And this may be achieved by sponge miR-574-5p. Our study expands new ideas for the management of sepsis ARDS |
| Limitations | 19 | Discuss limitations of the study, taking into account sources of potential bias or imprecision. Discuss both direction and magnitude of any potential bias | 12-13 | However, the specific molecular mechanism of the HOTTIP/miR-574-5p axis will be covered in the next studies. There are undeniable limitations to this study. First, this case-control study was conducted in a single center and the sample size was compelled by the available number of patients with sepsis. Second, APACHE III scores have recently been found to show higher differentiation in hospital mortality. However, we have used the earlier and more widely available APACHE II scores, this is a potential limitation of this preliminary study. The APACHE III score will be used for validation in subsequent multicentre in-depth studies with larger sample sizes. Furthermore, although we selected 103 overlapping genes, it is necessary to further investigate the mRNAs that act on the HOTTIP/miR-574-5p axis, which may be a key target for the treatment of ARDS in sepsis. Finally, the detection of HOTTIP levels in clinical practice may need to be performed in well-equipped hospitals, so its generalizability in clinical practice needs to be further explored. Nevertheless, our study broadens new perspectives for the diagnosis of sepsis, but further exploration of biomarkers that simplify the testing procedure and are cost-effective for clinical practice is needed. |
| Interpretation | 20 | Give a cautious overall interpretation of results considering objectives, limitations, multiplicity of analyses, results from similar studies, and other relevant evidence | 12-13 | However, the specific molecular mechanism of the HOTTIP/miR-574-5p axis will be covered in the next studies. There are undeniable limitations to this study. First, this case-control study was conducted in a single centre and the sample size was compelled by the available number of patients with sepsis. Second, APACHE III scores have recently been found to show higher differentiation in hospital mortality. However, we have used the earlier and more widely available APACHE II scores, this is a potential limitation of this preliminary study. The APACHE III score will be used for validation in subsequent multicenter in-depth studies with larger sample sizes. Furthermore, although we selected 103 overlapping genes, it is necessary to further investigate the mRNAs that act on the HOTTIP/miR-574-5p axis, which may be a key target for the treatment of ARDS in sepsis. Finally, the detection of HOTTIP levels in clinical practice may need to be performed in well-equipped hospitals, so its generalizability in clinical practice needs to be further explored. Nevertheless, our study broadens new perspectives for the diagnosis of sepsis, but further exploration of biomarkers that simplify the testing procedure and are cost-effective for clinical practice is needed. |
| Generalisability | 21 | Discuss the generalisability (external validity) of the study results | 10, 13 | elevated HOTTIP is a potential diagnostic biomarker for sepsis ARDS and can predict the onset of short-term mortality. And this may be achieved by sponge miR-574-5p. Our study expands new ideas for the management of sepsis ARDS.  Finally, the detection of HOTTIP levels in clinical practice may need to be performed in well-equipped hospitals, so its generalizability in clinical practice needs to be further explored |
| Other information | |  | | |
| Funding | 22 | Give the source of funding and the role of the funders for the present study and, if applicable, for the original study on which the present article is based |  | NO |

*Give information separately for cases and controls in case-control studies and, if applicable, for exposed and unexposed groups in cohort and cross-sectional studies.

**Note:** An Explanation and Elaboration article discusses each checklist item and gives methodological background and published examples of transparent reporting. The STROBE checklist is best used in conjunction with this article (freely available on the Web sites of PLoS Medicine at http://www.plosmedicine.org/, Annals of Internal Medicine at http://www.annals.org/, and Epidemiology at http://www.epidem.com/). Information on the STROBE Initiative is available at www.strobe-statement.org.
