# Supplementary material for: LncRNA HOTTIP as a diagnostic biomarker for acute respiratory distress syndrome in patients with sepsis and to predict the short-term clinical outcome: a case-control study
Source: BMC Anesthesiol. 2024 Jan 18;24:30. doi: 10.1186/s12871-024-02405-z (PMC10795278; doi:10.1186/s12871-024-02405-z)
Supplement: Supplementary file 2 — Additional file 2: Supplementary Figure. The flow chart diagram of patient inclusion. Supplementary Table 1. Identified 109 overlapping target genes of miR-574-5p in 5 databases. Supplementary Tabel 2. Overlapping genes between miR-574-5p predicted genes and sepsis, ARDS-related genes. Supplementary Table 3. Top 20 significant GO terms of the overlapped genes. Supplementary Table 4. Top 10 significant enriched pathways of the overlapped genes. [file 12871_2024_2405_MOESM2_ESM.docx]

**
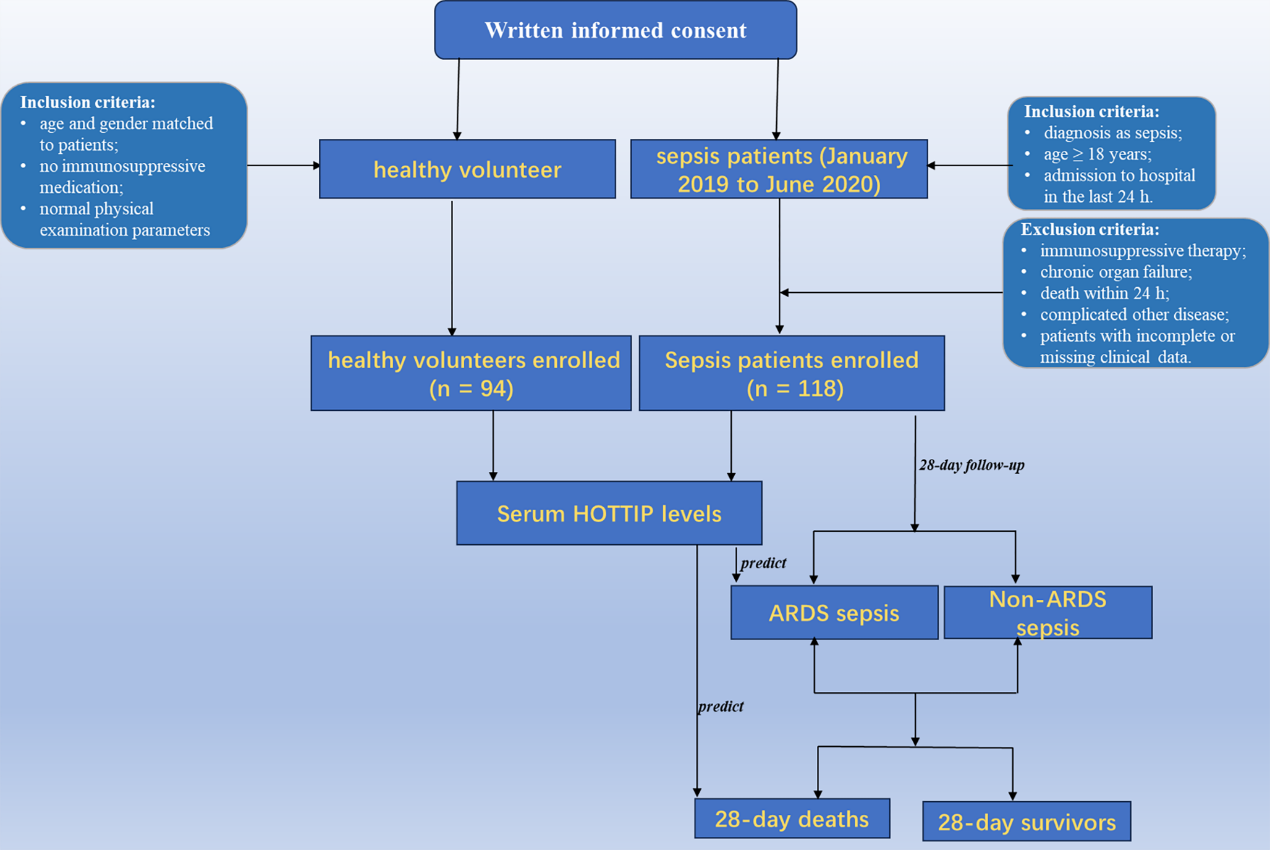
**

**Supplementary figure.** The flow chart diagram of patient inclusion.

**Supplementary Table：**

**Supplementary Table 1** Identified 109 overlapping target genes of miR-574-5p in 5 databases

| miRNA | Database | Overlapping genes |
| --- | --- | --- |
| miR-574-5p | miRWalk  EVmiRNA  ENCORI  CancerMIRNome  TargetScan | ABI2, AFF3, AMOTL1, ARHGAP35, ARHGEF9, ARPP19, ATMIN, BNC2, BRCC3, BRWD3, BVES, CAMK2N1, CAMK4, CCND1, CHRDL1, CLIC6, CNTNAP5, CREB3L2, DMD, DOCK1, DOK6, EFNB1, EHD3, FAM117B, FGF14, FGFRL1, FOXN3, FREM2, GABRB2, GFRA1, GLYR1, GOLIM4, GPBP1L1, HHIP, HS6ST3, IFFO2, IGF1, KAT6A, KCNJ6, KIAA1549L, KIF1B, LATS2, LCOR, LONRF1, LPP, LRRC58, LRRTM2, LYRM7, MAP7D1, MAPK10, MBNL3, MCM8, MGRN1, MRPS23, MSN, MYCBP, MYO5A, NAV1, NCDN, NCK2, NLGN3, NMNAT2, OLR1, PLAG1, PLEC, PLXDC2, PPP1R16B, PRKCB, RAB3C, RAD21, RALGAPB, RAP2C, RGS4, RGS5, ROCK2, SATB1, SCD, SLC2A12, SLC43A3, SLC7A11, SLC7A2,  SLC9A1, SMAD4, SNX2, SPATA6, SPRY4, SRGAP2, STOX2, STXBP6, SYNPO, TBC1D16, THRB, TMEM106B, TMEM130, TMEM184B, TNR, TOB2, TRIOBP, TTLL7, UBLCP1, UBN2, VPS36, VSNL1, ZBTB20, ZDHHC6, ZEB1, ZNF529, ZNF551, ZSWIM1 |

**Supplementary Tabel 2** Overlapping genes between miR-574-5p predicted genes and sepsis, ARDS-related genes

| miRNA | Disease Database | Overlapping genes |
| --- | --- | --- |
| miR-574-5p | CTD | ABI2, AFF3, AMOTL1, ARHGAP35, ARHGEF9, ARPP19, ATMIN, BNC2, BRCC3, BRWD3, BVES, CAMK2N1, CAMK4, CCND1, CHRDL1, CLIC6, CREB3L2, DMD, DOCK1, DOK6, EFNB1, EHD3, FAM117B, FGF14, FGFRL1, FOXN3, FREM2, GABRB2, GFRA1, GLYR1, GOLIM4, HHIP, HS6ST3, IGF1, KAT6A, KCNJ6, KIAA1549L, KIF1B, LATS2, LCOR, LONRF1, LPP, LRRC58, LRRTM2, LYRM7, MAP7D1, MAPK10, MBNL3, MCM8, MGRN1, MRPS23, MSN, MYCBP, MYO5A, NAV1, NCDN, NCK2, NLGN3, NMNAT2, OLR1, PLAG1, PLEC, PLXDC2, PPP1R16B, PRKCB, RAB3C, RAD21, RALGAPB, RAP2C, RGS4, RGS5, ROCK2, SATB1, SCD, SLC2A12, SLC43A3, SLC7A11, SLC7A2,  SLC9A1, SMAD4, SNX2, SPRY4, SRGAP2, STOX2, STXBP6, SYNPO, TBC1D16, THRB, TMEM106B, TMEM130, TMEM184B, TNR, TOB2, TRIOBP, TTLL7, UBLCP1, UBN2, VPS36, VSNL1, ZBTB20, ZDHHC6, ZEB1, ZSWIM1 |

**Supplementary Table 3** Top 20 significant GO terms of the overlapped genes

| Category | GO ID | Term | List | P-value |
| --- | --- | --- | --- | --- |
| BP | GO:0045446 | endothelial cell differentiation | 6 | 0.0000 |
| BP | GO:0010611 | regulation of cardiac muscle hypertrophy | 5 | 0.0000 |
| BP | GO:0014743 | regulation of muscle hypertrophy | 5 | 0.0000 |
| BP | GO:0003158 | endothelium development | 6 | 0.0001 |
| BP | GO:0061028 | establishment of endothelial barrier | 8 | 0.0001 |
| BP | GO:1901019 | regulation of calcium ion transmembrane transporter activity | 4 | 0.0001 |
| BP | GO:0110053 | regulation of actin filament organization | 9 | 0.0001 |
| BP | GO:0032956 | regulation of actin cytoskeleton organization | 5 | 0.0001 |
| BP | GO:0034764 | positive regulation of transmembrane transport | 7 | 0.0001 |
| BP | GO:1902903 | regulation of supramolecular fiber organization | 9 | 0.0001 |
| MF | GO:0017049 | GTP-Rho binding | 3 | 0.0002 |
| MF | GO:0017048 | Rho GTPase binding | 6 | 0.0002 |
| CC | GO:0005923 | bicellular tight junction | 5 | 0.0004 |
| CC | GO:0070160 | tight junction | 5 | 0.0005 |
| MF | GO:0005096 | GTPase activator activity | 7 | 0.0006 |
| CC | GO:0070160 | tight junction | 5 | 0.0005 |
| CC | GO:0043296 | apical junction complex | 5 | 0.0008 |
| CC | GO:0005911 | cell-cell junction | 9 | 0.0010 |
| MF | GO:0030695 | GTPase regulator activity | 7 | 0.0011 |
| MF | GO:0017016 | Ras GTPase binding | 8 | 0.0014 |

**Supplementary Table 4** Top 10 significant enriched pathways of the overlapped genes

| Category | ID | P value | Genes |
| --- | --- | --- | --- |
| Tight junction | hsa04530 | 0.0000 | AMOTL1/BVES/CCND1/MAPK10/MSN/RAP2C/ROCK2/SYNPO |
| Focal adhesion | hsa04510 | 0.0000 | ARHGAP35/CCND1/DOCK1/IGF1/MAPK10/PRKCB/ROCK2/TNR |
| cAMP signaling pathway | hsa04024 | 0.0002 | BVES/CAMK4/CREB3L2/HHIP/MAPK10/ROCK2/SLC9A1 |
| Glioma | hsa05214 | 0.0008 | CAMK4/CCND1/IGF1/PRKCB |
| Proteoglycans in cancer | hsa05205 | 0.0009 | CCND1/IGF1/MSN/PRKCB/ROCK2/SLC9A1 |
| Oxytocin signaling pathway | hsa04921 | 0.0016 | CAMK4/CCND1/KCNJ6/PRKCB/ROCK2 |
| Regulation of actin cytoskeleton | hsa04810 | 0.0016 | ABI2/ARHGAP35/DOCK1/MSN/ROCK2/SLC9A1 |
| Prostate cancer | hsa05215 | 0.0020 | CCND1/CREB3L2/IGF1/ZEB1 |
| AGE-RAGE signaling pathway in diabetic complications | hsa04933 | 0.0023 | CCND1/MAPK10/PRKCB/SMAD4 |
| Wnt signaling pathway | hsa04310 | 0.0025 | CCND1/MAPK10/PRKCB/ROCK2/SMAD4 |
